# Physical Activity Fragmentation and All-Cause Mortality in Hemodialysis Patients: Insights From Fitbit Study

**DOI:** 10.1016/j.xkme.2026.101304

**Published:** 2026-02-14

**Authors:** Armin Ahmadi, Subhasis Dasgupta, Maggie Han, Peter Kotanko, Rakesh Malhotra

**Affiliations:** 1 Division of Nephrology-Hypertension, Department of Medicine, University of California San Diego, San Diego, CA, USA; 2 San Diego Supercomputer Center, University of California San Diego, La Jolla, CA, USA; 3 Renal Research Institute, New York, NY, USA; 4 Department of Nephrology, Icahn School of Medicine at Mount Sinai, New York, NY, USA

To the Editor,

Physical frailty is highly prevalent among individuals with kidney failure requiring replacement therapy, often manifesting as reduced physical activity and impaired functional capacity.^1^ Low physical activity in dialysis patients has been linked with increased risk of hospitalization, cardiovascular events, and all-cause mortality.^2^ Although total daily activity is commonly assessed, less is known about whether the pattern in which activity is accumulated provides additional prognostic insight.

Activity fragmentation, defined as the ratio of active segments to total activity, offers complementary insight into how activity is accumulated throughout the day.^3^ Prior studies have linked greater activity fragmentation with poor physical functioning and increased frailty.^4^^,^^5^ Thus, activity fragmentation may reflect a distinct dimension of physiological and functional decline. The primary objective of this study was to examine the association between activity fragmentation and mortality risk in dialysis patients. The secondary objective was to explore the relationship between activity bout duration and mortality in dialysis patients.

Dialysis patients wore activity trackers (Fitbit Charge 5; Google, San Francisco, CA) between March 2018 and April 2020 with mortality data collected until December 2023. All participants provided written informed consent, and the study protocol was approved by the institutional review board (IRB 171917). Minute-by-minute activity data over a 7-day period was used to categorize each minute as active (≥10 steps/min) or sedentary (<10 steps/min). Consecutive active minutes were grouped into activity bouts to characterize the duration and continuity of movement. Activity fragmentation was calculated as the reciprocal of the average active bout duration representing the frequency of transitions from activity to inactivity. For example, if someone is active for an average of 4 minutes, their activity fragmentation would be 0.25 or 25%, meaning they transition activity to inactivity approximately every 4 minutes. Higher activity fragmentation indicates shorter, more frequent bouts of activity, whereas lower fragmentation reflects more continuous and sustained activity. In this study, valid wear time was defined as periods when the Fitbit activity tracker was worn and generated plausible activity data, which typically corresponded to waking hours. A valid wear day was defined as a day with >10 hours of valid wear time, consistent with prior literarture.^3^^,^^4^

Active bouts was further categorized by duration (1 to <5 minutes, 5 to <10 minutes, and ≥10 minutes). We averaged the time spent in each category across the 7-day wear protocol. Cox proportional hazard models were used to evaluate the associations between activity patterns and mortality risk, adjusting for demographic (age, sex, race/ethnicity), device wear time, and comorbid conditions (diabetes, hypertension, heart disease, and pulmonary disease).

We analyzed data from 85 dialysis patients with mean ± standard deviation age of 62 ± 13 years. Among participants, 45% were female, and 37 (44%) died during the follow-up period. The mean follow-up duration was 3.1 ± 1.6 years. When comparing baseline characteristics, the survivors at follow-up were predominantly male (62% vs 46%) and had a shorter dialysis vintage (3.9 vs 5.4 years) compared with those who died. The deceased group had a higher prevalence of comorbid conditions, including diabetes (86% vs 50%), hypertension (95% vs 77%), history of heart disease (81% vs 35%), and pulmonary disease (30% vs 21%), compared with those who survived.

Participants who died exhibited significantly greater activity fragmentation, with a mean fragmentation index of 47% ± 6% compared with 37% ± 8% in those who survived. Those who died also had a significantly higher proportion of short activity bouts (1 to <5 minutes: 51% ± 12% vs 35% ± 10%) and a lower proportion of long activity bouts (≥10 minutes: 22% ± 12% vs 36% ± 12%) compared with survivors (Table 1).

**Table 1:** Descriptive Physical Activity Metrics Stratified by Mortality Status

Variables	Participants		*P*
	Alive (n = 48)	Deceased (n = 37)	
Total daily hours spent physically active, h/d (SD)	3.9 (1.5)	3.1 (1.4)	0.01
Fragmentation index, % (SD)	37.2% (7.8%)	46.6% (6.4%)	<0.001
Daily activity spent in bouts, % (SD)			
1 to <5 min	35.4% (10.2%)	51.3% (11.6%)	<0.001
5 to <10 min	28.5% (8.4%)	26.6% (7.1%)	0.26
≥10 min	36.1% (11.7%)	22.1% (12.1%)	<0.001

*Note: P* values for continuous variables were derived using two-sample *t* tests comparing survivors and deceased participants. Percentages for activity bouts represent the distribution of total active time rather than total wear time; inactivity can be inferred as the proportion of time not spent in active bouts.

Abbreviation: SD, standard deviation.

In both the unadjusted and adjusted models, higher total physical activity amount was significantly associated with lower mortality risk (adjusted hazard ratio, 0.95 [per 1 h/d increase in total activity]; 95% confidence interval, 0.80-0.99). In the fully adjusted Cox proportional hazards model, a higher activity fragmentation index was significantly associated with increased mortality risk (hazard ratio, 1.5 [per 10% higher degree of fragmentation]; 95% confidence interval, 1.1-2.0). Similarly, a higher percentage of short activity bouts (1 to <5 minutes) was independently associated with greater mortality risk (hazard ratio, 1.3 [per 10% higher proportion of total active time spent in short bouts]; 95% confidence interval, 1.1-1.7). However, time spent in 5 to <10 minutes or ≥10 minute bouts were not significantly associated with mortality (Table 2).

**Table 2:** Hazard Ratios for Total Physical Activity, Activity Fragmentation, and Time Spent in Activity Bouts of Varying Lengths in Relationship to Mortality

Variables	Hazard Ratio		
	Unadjusted	Model 1	Model 2
Total time physically active^a^	0.92 (0.74-0.97)	0.93 (0.77-0.95)	0.95 (0.80-0.99)
Activity fragmentation^b^	1.80 (1.18-2.21)	1.78 (1.20-2.14)	1.54 (1.08-2.04)
Activity spent in bouts^c^			
1 to <5 min	1.49 (1.12-1.72)	1.40 (1.14-1.75)	1.32 (1.08-1.66)
5 to <10 min	1.05 (0.80-1.51)	1.04 (0.81-1.54)	1.12 (0.95-1.42)
≥10 min	0.88 (0.72-0.99)	0.87 (0.70-1.18)	0.94 (0.72-1.13)

*Note:* Each predictor (total time physical activity [hr/day], activity fragmentation, and activity time spent in bouts [%]) was modeled separately in individual Cox regression models. Model 1: Adjusted for baseline age, sex, and race/ethnicity. Model 2: Model 1 + device wear time, diabetes, hypertension, heart disease, and pulmonary disease.

a Hazard ratios for total physical activity are per 1 h/d increase.

b Hazard ratios for activity fragmentation are scaled per 10% higher degree of activity fragmentation (ie, shorter, more interrupted activity bouts).

c Hazard ratios for activity spent in bouts (1 to <5 min, 5 to <10 min, ≥10 min) are per 10% higher proportion of total active time spent in each bout category.

In this study, we reported that lower total physical activity amount and fragmented daily physical activity, especially short bouts of 1 to <5 minutes, were strongly linked to increased mortality among dialysis patients, independent of comorbid conditions. These findings suggest that not only the quantity but also the pattern of physical activity provides prognostic information in this high-risk population.

Fragmentation of activity may reflect underlying physiological characteristics that may not be fully captured by total step count or duration alone. The use of wearable activity monitors allowed for granular assessment of real-world activity behavior, providing objective insights into functional status beyond traditional clinical measures.^6^ In line with previous findings from the Baltimore Longitudinal Study of Aging, our results support the utility of activity fragmentation as a sensitive phenotypic marker of deteriorating physical function and mortality.^3^^,^^7^ Identifying patients with highly fragmented activity could inform targeted interventions and tailored exercise prescriptions to improve physical resilience and outcomes. Future studies are warranted to assess whether longitudinal changes in activity fragmentation predict morbidity and mortality and whether interventions aimed at promoting longer, sustained bouts of activity can improve survival and quality of life in dialysis patients.

Limitations of this study include the observational design, which precludes causal inference, and potential residual confounding by unmeasured factors such as depression, functional capacity, or socioeconomic status. Additionally, data were collected during the COVID-19 pandemic, which may have influenced activity behaviors. The lack of GPS or contextual data also limits our ability to distinguish indoor from outdoor activity, which may affect interpretation of activity fragmentation.

In conclusion, both the amount and fragmented pattern of physical activity are important predictors of mortality in dialysis patients. Activity fragmentation, as captured through wearable devices, may serve as a clinically meaningful marker of physical vulnerability and a modifiable target for intervention.
